# Exploring the Nexus of Feeding and Processing: Implications for Meat Quality and Sensory Perception

**DOI:** 10.3390/foods13223642

**Published:** 2024-11-15

**Authors:** Sandra S. Q. Rodrigues, Ana Leite, Lia Vasconcelos, Alfredo Teixeira

**Affiliations:** CIMO, LA SusTEC, Instituto Politécnico de Bragança, Campus de Santa Apolónia, 5300-253 Bragança, Portugal; anaisabel.leite@ipb.pt (A.L.); lia.vasconcelos@ipb.pt (L.V.); teixeira@ipb.pt (A.T.)

**Keywords:** feeding, processing, meat quality, sensory perception, consumers, sustainability

## Abstract

The intrinsic quality of meat is directly related to muscle and fat tissues. Factors such as the rate and extent of anaerobic glycolysis affect muscle pH, influencing the meat’s color, water holding, and texture. Postmortem anomalies can result in deviations from this intrinsic quality. The animals’ diet plays a crucial role in meat quality. Specific nutrients, such as proteins, vitamins, and minerals, affect meat’s texture, flavor, and juiciness. Feeds rich in omega-3 fatty acids can improve the sensorial quality of meat. Meat processing and methods such as aging, marinating, and cooking affect the texture, flavor, and juiciness, which can be evaluated by specific equipment or trained or untrained consumers. This comprehensive review investigates the relationship between animal feeding practices and meat processing techniques and their combined impact on meat quality and sensory perception. By synthesizing recent research, we explore how various feeding protocols (including diet composition and feed additives) and processing methods shape meat products’ nutritional value, texture, flavor profile, and overall consumer appeal. Understanding this nexus is crucial for optimizing meat quality while ensuring sustainability and safety in the food supply chain.

## 1. Introduction

Meat and meat products provide essential high-quality proteins and easily absorbable micronutrients, including vital vitamins and minerals, which are crucial for a balanced human diet [1]. Quality is a subjective and dynamic concept but is usually accepted as “the product charactessristics that correspond to the consumers expectations”. The quality and sensory perception of meat and meat products are critical factors that influence consumer preferences and market success [2,3]. As the global demand for high-quality meat products continues to rise [4,5], understanding the intricate relationship between feeding practices and processing technologies that can influence meat and meat product quality, becomes increasingly important. This revision paper delves into the multifaceted interactions that shape meat quality from farm to fork.

The paper is structured around four key themes. First, it examines the impact of diet composition and feed additives or supplements on meat quality parameters. This section explores how various diet compositions, and the inclusion of feed additives can influence meat quality attributes such as physicochemical characteristics, sensory attributes (tenderness, juiciness, flavor), and nutritional value by analyzing the results obtained by various authors [6,7,8]. By exploring recent research and advancements, the paper highlights the pivotal role of feeding strategies in enhancing meat quality.

Next, the paper investigates advances in processing technologies and their effects on meat preservation and sensory characteristics. This part focuses on the latest innovations in meat processing technologies, including methods for preservation, and the enhancement of sensory characteristics, including thermal [9,10,11] and non-thermal processes [12,13,14], as well as the use of chemical or bio-preservatives [15]. The discussion centers on how these technologies can extend shelf life, maintain freshness, and improve the overall eating experience.

The third theme explores the interplay between feeding strategies [16] and processing [11] for enhanced meat safety and sustainability. It discusses the synergistic effects of feeding strategies and processing techniques on meat safety and sustainability, emphasizing how integrated approaches can mitigate risks, reduce environmental impact, and promote sustainable meat production practices.

Finally, the paper addresses consumer perception related to meat and meat products. This section examines how different feeding and processing practices influence consumer attitudes and preferences [17,18,19]. By analyzing market trends and consumer feedback, the paper provides insights into how quality perceptions are shaped and how they can be aligned with industry practices.

By synthesizing current research and industry practices, this paper aims to provide a comprehensive overview of the nexus between feeding and processing in the context of meat quality and sensory perception. The goal is to offer valuable insights for researchers, industry professionals, and policymakers to enhance meat quality, safety, and sustainability.

**Figure 1:**
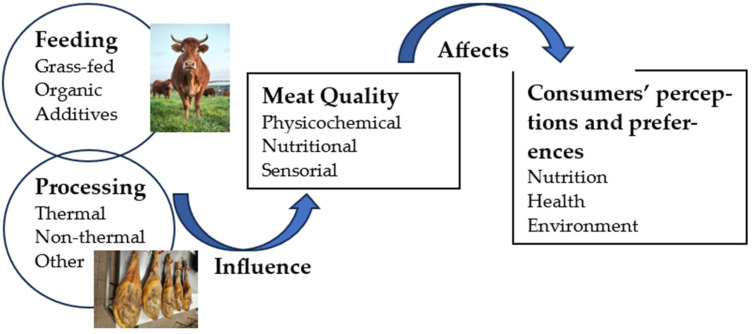
Diagrammatical summary.

Figure 1 represents a diagrammatical abstract of the present paper.

## 2. Impact of Diet Composition and Feed Additives on Meat Quality Parameters

Many factors can influence growth, carcass yield, and meat quality. Breed, sex, genetics, feed, age, and slaughter weight, among others, are critical factors that must be considered in management practices. For this reason, meat producers must identify these factors to know the quality standards. In this way, the producer will have the tools to apply the appropriate management practices to define the type of animal needed to meet the end consumer’s demands. Feeding practices can significantly impact several meat quality traits, from carcass composition and, therefore, the commercial value to the organoleptic quality and nutritional value of the meat and derived products. Among animal breeds, the introduction of a specific nutritional strategy has one main objective. On the other hand, producers should also consider some economic issues. The meat sector needs to study the raw materials to make them more economical, more sustainable, and able to provide physicochemical, nutritional, and organoleptic qualities in meat and its derivatives. Partial replacement of feed with by-products can help reduce costs and increase the food chain’s sustainability. The introduction of fats from other food sources increases the energy value (improving animal growth) and provides essential fatty acids and fat-soluble vitamins [6,7]. Changing the fatty acid profile by reducing the saturated fatty acid content and increasing the unsaturated fatty acid content is desirable because it increases the nutritional value and meets the needs of consumers looking for leaner, healthier meats [20]. However, animal feeding practices must consider other important factors when altering the lipid profile. Meat with higher levels of unsaturation is more likely to be oxidized, making it more susceptible to microbial oxidation and defects in color, texture, and flavor. The dietary nutrient composition of the animals’ diet, such as the protein/energy ratio, affects the carcass composition [21]. Table 1 shows the influence of the different types of food that are introduced into the animal’s diet.

The introduction of by-products into an animal’s diet has an initial effect on the carcass conformity, digestibility, and the animal’s immunity. All these factors can be related to the quality of the meat and consequently to the quality of its processed products. In the studies described above, we can see the influence of the addition of various by-products to the animal’s diet on the animal, the carcass, and consequently on the quality of the meat and its derived products.

Concerning lamb meat, the use of by-products of the animals, and the use of by-products in the animal´s diet have made it more acceptable to consumers. It is important to note that the introduction of these by-products did not negatively influence the animal´s growth or the conformity of the carcass. The proportion of polyunsaturated fatty acids in the total intramuscular lipids was affected by the dietary treatment [23]. The PUFA was higher from animals receiving feed supplemented with linseed or with olive cake and linseed, compared to animals fed the control diet [23].

Concerning the pork species, several by-products were used, such as shredded acorns [24], insects (*Tenebria molitor*) [25], tomato pomace [26], olive cake [27], vitamin D2-enriched mushroom powder [28], bergamot pulp [29], olive pomace acid oil [6]. The use of by-products did not harm the animals’ performance [26] and there was an improvement in immunity and the prevention of infection by pathogenic bacteria [25]. Although these effects are more related to animal welfare, they will influence the quality of the meat and its processed products. The introduction of olive cake into the diet of pigs did not affect meat quality or fat quality. However, it did affect muscle length [27]. The introduction of vitamin D and Bergamot pulp [28,29] increased antioxidant capacity and oxidative stability.

For other species, such as chickens, ducks and rabbits, the introduction of by-products (plant polyphenolic compounds [30], diets supplemented with proteins, fats, and carbohydrates [31], garlic leaves [32], sorghum grain [33], hempseed meal [34], marine macroalgae products [37]) into animal feed has also been beneficial, without negatively affecting the animal, the carcass or consequently the quality of the meat and processed products.

## 3. Advances in Processing Technologies and Their Effects on Meat Preservation and Sensory Characteristics

Since ancient times, humans have tried to preserve their food using techniques that allow them to extend the shelf life. Without these preservation methods, degradation, microbiological activity, chemical and enzymatic reactions, and physical changes are inevitable. Even using various methods, including refrigeration, freezing, curing, smoking, thermal processing, canning, dehydration, chemicals, and applying pressure, there can be difficulties in eliminating certain harmful microorganisms.

In this sense, some of the more traditional methods of food preservation, such as dehydration, smoking, brining, canning, fermentation, and refrigeration, have been replaced or complemented with other more innovative preservation techniques (chemical or bio-preservation and non-thermal). Below are just a few of the technologies that have been applied, among the most “in vogue”, intending to balance sensory quality, nutritional value, and microbial safety.

### 3.1. Thermal Processing

#### 3.1.1. Dry-Aging

In the global food processing sector, drying remains an essential technique. Wet-aging (with vacuum packing) and dry-aging (without vacuum packing) are the two most popular methods of postmortem aging.

Dry-aging is a traditional method used to remove moisture from food materials to improve cut meats’ palatability, especially regarding taste perception, softness, and marbling [39]. Various techniques are used to dry meat, including vacuum drying, ultrasonic drying, freeze-drying, microwave drying, heat pump drying, pulsed electric field drying, and refractance window drying [40]. Frequently used on premium meat, dry-aging requires highly regulated ambient temperature, relative humidity, and airflow conditions [41].

Some changes in the functional and sensory attributes of food materials are recognized as negative, regarding the quality of the final product [42,43,44], since the type of dryer, the drying conditions, and the composition and physical properties of the raw food material influence the type of final product that will be obtained.

Due to the concentration of important nutrients during dry-aging, such as proteins, fats, and minerals, chewing dry-aged meat releases juicy, fatty substances that enhance flavor, nuttiness, umami, and other sensory qualities [45,46]. Therefore, dry-aging meat is well known to improve meat tenderness and flavor. Recently, a study by Hwang et al. [47], applied to pork belly and shoulder blade cuts, found that dry-aging leads to greater redness of the meat and lipid oxidation due to unprotected storage during aging, but greater tenderness and greater protein degradation, which results in an increase in water holding capacity and a decrease in shear force.

However, from a microbiological point of view, although dry-aging generally reduces levels of Salmonella and *E. coli* O157:H7, it is not effective in combating *Listeria monocytogenes* and *Yersinia enterocolitica*, which can multiply in uncontrolled conditions [48].

So, the dry-aging process continues to be part of a range of meat processing and preservation technologies that have a major impact on sensory and nutritional qualities [11].

#### 3.1.2. Sous-Vide Cooking

Sous-vide cooking is a method of sealing food in a plastic bag that maintains its freshness and submerging it in a water bath kept at a carefully regulated low temperature (53–81 °C) for a long period of time (LTLT) (10–48 h) [49]. This technology has been widely used in catering, food retail, and the health food market [50,51]. With vacuum sealing, this method allows heat to be transferred efficiently from water (or steam) to food, increasing shelf life by eliminating the risk of recontamination during storage; it also inhibits unpleasant flavors from oxidation and prevents evaporation losses of flavor volatiles and moisture during cooking [52]. This method seeks to maximize the consistency and palatability of meat and meat products [53,54].

Although sous-vide cooking offers advantages for preservation, it differs from conventional techniques such as canning or drying. Proper food handling, storage, and pasteurization are essential to ensure food safety. Moreover, other techniques, such as marinating, can be used to improve the result of the cooking of meat products, for example, beef’s tenderness, flavor, and juiciness [55]. Another study by Gomez et al. [56] emphasizes the feasibility of using the combination of marinating and sous-vide cooking techniques to yield new ready-to-eat products with a high protein content from meat without negatively affecting quality characteristics.

However, microbial contamination remains a concern with low-temperature cooking [57], which requires another complementary technique like bio-preservatives, for example.

#### 3.1.3. Freezing

A thermal method increasingly being used to preserve fresh meat quality and prolong shelf life is the freezing method [9]. The most used are ultrarapid freezing, vacuum immersion cooling, hydro fluidization freezing, impact freezing, impingement freezing, miscellaneous advanced freezing, electrostatic assisted freezing, pressure displacement freezing, magnetic resonance-assisted freezing, and acid electrolyzed water associated with high hydrostatic pressure, etc. [58]. There is considerable evidence that these methods inhibit microbial growth and reduce enzyme activity [15]. There are key factors for successful freezing methods, such as correct packaging to avoid freezer burn, temperature stability, and, in some cases, blanching before freezing, as this is essential to maintain the color, taste, and texture of foods. However, the organisms will be deactivated rather than killed and, most of the time, may be activated when the frozen product is thawed. In that case, natural convection thawing (NCT) generally yields better results than running water thawing [59].

Also, there are two other techniques not mentioned above, such as vacuum packing with freezing and individual quick freezing (IQF), which are well-established in the meat industry and known for their practicality, scalability, and effectiveness. These methods are crucial for their cost-effectiveness, reliability, and compatibility with industrial processes. Studies on beef and pork have demonstrated that IQF minimizes quality deterioration, reduces drip loss, and maintains better cell integrity [59]. Authors like Beltran and Belles [60] revealed that consumers could not see contrasts between fresh and frozen meat regarding texture and flavor. However, freezing has also been found to cause a series of physical and biochemical changes in muscle foods, including the formation of ice crystals, concentration of solutes, alteration of ionic strength and pH, freezer burn, discoloration, lipid oxidation, and protein denaturation [61]. Additionally, freezing can increase drip/thaw and cook losses [58]. It is, therefore, essential to relate and intercalate this method with other types of processing to make the most of its application in the food industry.

### 3.2. Non-Thermal Processing

#### 3.2.1. High Pressure Processing

High hydrostatic pressure (HHP) technology is used to preserve high-quality products to maintain nutritional value [62].

A meat preservation technology using high-pressure processing is an alternative to traditional thermal methods of inactivating microorganisms [63] as it allows microbiological safety without significantly increasing the temperature [58,64]. In addition, HHP makes it possible to extend the shelf life while maintaining the organoleptic characteristics (i.e., taste, smell, color, texture) and natural nutritional values of the raw material without chemical additives [63,65]. To preserve meat products, pressure should be applied in the 400–600 MPa range, and the duration of action should be around 3 to 7 min. However, bacterial resistance to high pressure depends on the type and strains of bacteria [58]. The highest sensitivity to high pressure is shown by cells of Gram-negative bacteria, which die at pressures above 100 MPa [66].

#### 3.2.2. Irradiation

The application of gamma radiation to meat products is a non-thermal technology used to destroy pathogenic and spoilage microbes in food products [67] and increase their shelf life [68]. Food can be irradiated with ionizing radiation, such as gamma rays, X-rays, or high-energy electrons [69]. Gamma radiation allows the microbial destruction of food without substantially increasing the temperature of the food, generating the desired action only during food irradiation. Red meat products can be colored to taste better, have lower sodium nitrite content, and limit microbial growth by using this method. However, because radiation can accelerate lipid oxidation, produce free radicals and hydrogen peroxide in the presence of oxygen, and destroy compounds like antioxidants and carboxylic acids, this treatment is not advised for foods high in fat [70,71,72]. In a study by Silva et al. [67], gamma radiation treatment ensured microbiological safety and improved the organoleptic characteristics of cooked hams. However, in another study carried out by Feng et al. [73], the authors found that uncured and irradiated cooked turkey meat remained more sensitive to lipid oxidation than cooked turkey products. Smaller amounts of volatile compounds with an unpleasant odor were observed in the cured meat samples than in the uncured samples [73]. Some strategies have been applied to counteract this oxidation using irradiation. An example of this is the addition of bioactive compounds such as essential oils or their encapsulated active ingredients and the use of combined treatments (mild heat treatment and modified atmospheric packaging) since the relative bacterial sensitivity increases, allowing for a reduction in the dose of irradiation required to maintain food preservation [74,75].

#### 3.2.3. Plasma Technology

Plasma technology is a non-thermal technology that essentially uses cold plasma (CP) to reduce the counts of pathogenic and spoilage microorganisms in foodstuffs [76,77,78]. This technique is perfect because it can be applied to the disinfection of air, water, and food surfaces and processes the materials without causing any damage to living tissue [79]. It also enables decontamination at low temperatures, at low pressures, or even in a vacuum with little energy expenditure, which implies a low cost of use for industry [80]. This method is suitable for fresh food products such as milk, fruits, vegetables, and meat products, as it has no negative impact on nutritional and sensory characteristics. A study by Yong et al. [81] observed that plasma treatment reduced the number of bacteria and molds in packaged dried meat without major differences in some sensory parameters, including unpleasant odor. In another study by Moutik [82], the technique had a minimal effect on poultry meat’s color, pH, and water-holding capacity, making it an attractive alternative to traditional processing methods. Additionally, Jayasena et al. [83] believe it has potential applications in meat curing, as it can generate nitrite, an essential component of the curing process.

However, there are the disadvantages of requiring qualified personnel, high-cost investment in process-specific equipment [84], and challenges such as lipid oxidation and modification of the packaging material [78,83]. For industrial purposes, cold plasma treatment is not certified as an antimicrobial technology, so future studies are needed in relation to oxygen-reduced atmospheres and biopolymeric materials [85].

### 3.3. Other Forms of Food Preservation

#### Chemical and Bio-Preservatives

More and more natural antimicrobial agents, antioxidants, essential oils and some colorants are being used to replace other active compounds to extend the shelf life of food products, including meat. Lettuce, spinach, carrot and ginger extracts, rosemary and oregano oils, spices such as garlic and pepper, chitosan, sodium lactate, synthetic antioxidants, etc., are promising sources for food preservation. Some of them naturally contain nitrates capable of replacing existing nitrites and nitrates; others can inhibit the growth of bacteria, yeasts, and molds [86,87,88,89,90]. A proposal was made [91] to use *Bougainvillea spectabilis* as an innovative additive in cooked ham, aiming to replace conventional nitrite salts. This study evaluates the impact of different drying methods (air-drying, foam-mat drying, and oven drying) on the preparation of bougainvillea powder and the effect of these methods on the physicochemical and sensory properties of the ham, including antioxidant characteristics. Incorporating bougainvillea powders in the ham formulation improved the sensorial attributes and consumer overall acceptance, even after 8 weeks of cold storage at 4 °C.

According to Das et al. [92], mustard oil, as opposed to soybean and flax seed oils, can be utilized for meat marinating and preservation to increase the shelf life of chilled meat. However, it is necessary to know how to apply and use essential oils as natural antimicrobial agents for meat and meat products since they produce intense aromas and flavors that can affect the sensory quality of these products [93]. Another interesting study conducted by Ali et al. [94] demonstrated that bee honey contains some antioxidants that can reduce the microbial load and increase the shelf life of meat and meat products. Also, synthetic antioxidants, such as butylated hydroxyl anisole (BHA) and butylated hydroxyl toluene (BHT), are applied to meat to stop lipid oxidation and prevent the appearance of unpleasant odors and flavors [95].

Montaño-Sánchez et al. [96] reported the effect of increased physicochemical and microbiological qualities of born-in pork samples during storage by using chitosan in combination with green tea water extract. Gómez et al. [11] argue that introducing binding additives into meat, such as phosphates, thickeners, starches, sodium alginate, etc., helps to stabilize the emulsion and increase the water-holding capacity of these products.

The main findings in various studies on processing techniques and their effect on meat quality are presented in Table 2.

## 4. Interplay Between Feeding Strategies and Processing for Enhanced Meat Safety and Sustainability

The growing global demand for meat necessitates the development of efficient strategies to ensure product safety, sustainability, and quality. Enhancing meat quality through innovative feeding practices and processing technologies is critical in addressing these challenges. In particular, the interaction between feeding strategies and processing techniques is crucial in improving meat safety, sustainability, and overall quality, requiring a balanced approach that considers both aspects simultaneously.

Feeding strategies, including animal diet composition, growth conditions, and the use of feed additives, have significant effects on animal health, growth rates, and the safety of the final product. Today, innovative feeding approaches focusing on improving meat quality are essential. These strategies often involve using plant by-products, which can positively impact global food security and reduce environmental impact [97]. Furthermore, feed or nutritional management can enhance sustainability by improving animal productivity and mitigating environmental impacts [98]. However, the benefits of feeding strategies are closely tied to environmental and physiological conditions under which livestock are raised, highlighting the complex relationship between the two [99]. Elements such as housing, stocking density, and climate control can affect stress levels, impacting meat quality [100,101,102]. In this context, optimal growth conditions prioritizing animal health and welfare become crucial for the animals’ well-being and for producing higher-quality meat.

The diet provided to animals significantly influences the quality of the meat, including its texture, flavor, and safety, while contributing to sustainability [16,27,103,104]. This highlights how feeding practices can serve as a foundation for improving meat quality. However, these efforts must be complemented by effective processing techniques to ensure the desired outcomes. At the same time, processing techniques—such as slaughtering, packaging, and preservation—are equally important in ensuring meat safety, extending shelf life, and improving overall quality [11,15]. It is clear that feeding and processing practices must work together to achieve the best results.

The global demand for healthier and more sustainable meat products has led to increased interest in feed additives that can enhance the nutritional value of meat. Feed additives are substances added to animal feed to improve the nutritional content, health, and productivity of livestock. Moreover, specific feed additives can also enhance the nutritional value of the meat, making it healthier and more sustainable. This includes additives that reduce harmful substances and boost the nutritional profile of the final product [105,106]. In this way, feeding strategies affect animal growth and health and contribute to the long-term sustainability of meat production by improving the final product’s quality and safety. Probiotics are live microorganisms that, when administered in adequate amounts, confer a health benefit on the host animal. They can improve gut health, enhance nutrient absorption, and reduce the incidence of diarrhea in livestock. Probiotics can strengthen the immune system and reduce the need for antibiotics, leading to healthier animals and safer meat for consumption [107]. By reducing the reliance on antibiotics, probiotics contribute to the sustainability of livestock farming by minimizing the risk of antibiotic resistance [108]. Also, the use of organic acids [109] as well as some essential oils [110,111] can reduce the incidence of bacterial infections, improve gut health, reduce inflammation, and enhance the immune response in livestock, contributing to more sustainable livestock farming by reducing the risk of antibiotic resistance. The use of amino acid supplementation [112] or enzymes [113] can improve growth rates, feed efficiency, nutrient absorption, reduce the incidence of gut disorders, and enhance the overall health of livestock. By optimizing protein utilization, amino acid supplementation can reduce the environmental impact of livestock farming by decreasing nitrogen emissions [114]. So, the strategic use of feed additives will become increasingly important in meeting consumer expectations and environmental sustainability goals.

On the other hand, achieving sustainability in meat production requires a holistic approach that integrates feeding and processing practices. Feeding strategies, such as sourcing feed locally and reducing reliance on non-renewable feedstocks, can lower the environmental footprint of meat production. However, focusing on energy-efficient processing methods that reduce waste and improve the industry’s overall sustainability is equally important. The study from Parlasca and Qaim [5] emphasizes the need for sustainable practices throughout the food system, from farm to fork, due to the significant economic, social, and environmental impacts of meat and livestock production. To develop sustainable meat consumption patterns, the authors advocate a holistic approach that considers all sustainability dimensions—economic, social, environmental, health, and animal welfare. The authors call for policies and incentives to promote sustainable practices, engaging producers and consumers in the transition towards sustainability, and stress the importance of understanding sustainability across the entire lifecycle of meat products.

Incorporating certain agro-food co-products into animal diets promotes a circular economy by reducing waste and environmental impact. This practice not only supports normal animal growth [103] but also enhances meat quality [104,115], illustrating the close relationship between resource management and product quality. Additionally, sustainable practices in meat processing—such as reducing waste and energy consumption—further enhance the sustainability of the meat supply chain [116]. However, for these practices to be effective, they must be communicated effectively among stakeholders, including producers, retailers, consumers, and industry personnel. Collaboration is essential for adopting sustainable practices that optimize meat safety and quality [98].

The use of sustainability indicators and impact assessments in both feeding strategies and processing methods can guide the development of informed approaches that contribute to sustainable food systems. These indicators provide measurable benchmarks that can help the industry address sustainability challenges more effectively. However, significant challenges remain in ensuring sustainability across food chains, given the complexity and variability of global food systems. As Knorr et al. [117] emphasize, establishing harmonized sustainability indicators is a key requirement for objectively measuring sustainable systems. Additionally, the design of sustainable processes and engineering systems should incorporate emerging technologies that reduce water and energy consumption. Such integration ensures that all aspects of meat production—from feeding to processing—are aligned with sustainability goals.

In conclusion, by integrating these elements—feeding strategies, processing techniques, and sustainability metrics—the meat industry can achieve significant improvements in safety, sustainability, and overall quality. This will not only benefit consumers through healthier diets but also contribute to the development of a more sustainable global food system.

## 5. Consumer Perception Related to Meat and Meat Products from Different Feeding and Processing Practices

When selecting a meat product, how consumers perceive the characteristics of these products significantly shapes their attitude [118]. The main factors influencing consumer perception of meat and meat product quality can be categorized into intrinsic and extrinsic attributes [2,17,18]. Intrinsic attributes include the sensory characteristics of meat, which are among the key factors that shape consumer attitudes [119]. Taste is the characteristic most frequently associated with food preference, and the flavor profile plays a crucial role in determining consumer choice [120]; texture, mainly softness, and tenderness, are positively associated with consumer evaluation [121]; and the visual appeal of meat, particularly its color, significantly influences consumer choices, as just a small color variation can be detected by consumers [122]. Additionally, the nutritional value is very important, as consumers consider the health benefits of food’s nutritional composition, meat included [123].

Extrinsic attributes, external cues that consumers use to assess quality, include price and credence attributes (sustainability, animal welfare and origin, and label information). Consumers’ willingness to pay often correlates with their perception of quality; higher prices are typically associated with better quality [119,124]. Consumers increasingly worry about how meat is produced, considering environmental impact, social responsibility, ethical practices, and animal welfare [125,126]. Therefore, information on product labels [127], including certifications related to animal welfare [128] and health benefits [129], can shape consumer expectations [130].

Individual lifestyle choices and preferences play a significant role in consuming meat and meat products. For instance, urban consumers may prioritize convenience, while rural consumers might prefer traditional products. Social influences, media representation, and marketing strategies also affect consumer perceptions and can significantly impact consumer attitudes [2].

Overall, consumer perception of meat quality is multifaceted, involving a combination of sensory attributes, external cues, personal preferences, and broader societal influences. Consumer preferences and perceptions regarding meat products can vary significantly based on feeding and processing approaches, given that both can affect the aforementioned factors.

### 5.1. Feeding Approaches

Several research studies have investigated how different feeding systems influence consumer perceptions about meat quality in recent years. These studies reveal a nuanced understanding of how feeding practices impact the characteristics of meat and, subsequently, consumer preferences.

Consumers often perceive grass-fed meat as healthier and more natural than grain-fed meat. Grass-fed meat is associated with better animal welfare and environmental benefits, which can influence purchasing decisions [131]. One notable focus has been on lamb production, where extensive feeding systems, such as pasture-based feeding, enhance certain meat quality traits that align with consumer preferences for healthier and environmentally sustainable products. Lambs raised on high-quality pastures tend to have higher omega-3 fatty acid content, which is desirable for health-conscious consumers [132]. Additionally, research on restricted grazing and indoor supplementary feeding in lambs demonstrated that feeding systems significantly affect meat quality, with different grazing times producing variations in meat flavor and fat content, catering to diverse consumer tastes [133]. Studying the effect of feeding meat goats with sericea lespedeza (SL, *Lespedeza cuneata*) hay, a forage with highly condensed tannins, which are believed to negatively impact animal performance by reducing digestibility and voluntary intake, Lee et al. [134] found an increase in body weight compared to Bermuda grass hay without altering chemical composition and meat quality, and cooked chops flavor volatiles, suggesting that sericea lespedeza could serve as a cost-effective forage alternative to more expensive options. On the other hand, the lipid profile of the lamb meat was less favorable to consumer health when the animals were subjected to 60% feed restriction [135].

Organic meat is perceived to be superior in quality and safety, with higher health consciousness and environmental concerns driving consumer preferences [136]. Additionally, there is a growing preference for organic meat, which is perceived as free from antibiotics and hormones. Consumers paying a premium for organic products often cite health and environmental concerns [19]. A systematic literature review and meta-analysis on the composition differences between organic and conventional meat [137] revealed that organic livestock production has the potential to alter fatty acid profiles and other compositional parameters. These changes, such as increased n-3 polyunsaturated fatty acids (PUFA), may offer nutritional benefits. Additionally, even without mentioning organic, comparing supplementation (pasture-only vs. pasture plus 2% soy hull) on lambs’ meat quality, Lee et al. [138] stated that pasture-only lambs had healthier fatty acid profiles than those from supplemented lambs. To address the limitations in the current evidence base, it is crucial to conduct additional studies [137]. If these studies confirm nutritionally significant composition differences and/or link them to specific agronomic practices (such as high-forage diets), further dietary intervention or cohort studies would be justified. These studies could then identify the impact of consuming meat with different compositions resulting from organic production or particular agronomic practices.

In pork, nursery feeding programs were examined to understand their impact on subsequent growth performance and meat quality. It was found that while simpler diets (primarily consisting of corn and soybean meal, with whey and fishmeal included only in phase one of three phases) during the nursery phase could reduce costs, they did not adversely affect carcass or meat quality by market weight when compared with complex diets (containing highly digestible, complex animal protein sources, e.g., whey, fishmeal, spray-dried blood meal, and blood plasma, acidifiers, feed flavoring, and more easily digestible cereal grains). This suggests that cost-effective feeding strategies can be developed without compromising meat quality, an important consideration for producers and consumers [139]. Still, in pigs, no significant differences were found in the consumer’s evaluation of dry-cured loins from animals fed with peas or soy [140]. However, if there were differences due to feeding, they could have been masked by the use of additives and the changes in physicochemical characteristics produced during processing.

Studies on poultry have highlighted the importance of appearance and texture as primary quality attributes influencing consumer selection. Feeding practices, such as the introduction of distillers dried grains with solubles (DDGS), showed minimal impact on poultry meat quality, indicating that such dietary changes might be adopted without affecting consumer perception of meat quality [141]. Furthermore, feeding strategies that alter the timing and proportion of feed intake, especially under stress conditions, were found to improve growth and meat quality in turkeys, suggesting potential for optimization in feeding schedules to enhance product quality under varying environmental conditions [142].

These studies underscore the interconnectedness of feeding practices, meat quality, and consumer perceptions. They highlight the importance of aligning production methods with consumer demands for quality, health benefits, and sustainability to ensure market success.

### 5.2. Processing Approaches

In the past years, several research studies have explored consumer perceptions regarding the effects of meat processing on quality. These studies span various aspects of meat processing, from traditional methods to innovative technologies and their impact on sensory and nutritional quality.

One significant area of research has been examining sensory and nutritional aspects of meat processing and preservation technologies. This includes methods such as dry aging, dry curing, high hydrostatic pressure (HHP), conventional cooking, sous-vide cooking, and even 3D printing, as well as chemical methods like fermentation, smoking, curing, and marination. These studies emphasize the balance between retaining nutritional value and enhancing sensory quality, an area of great interest to consumers who prioritize both taste and health benefits in their meat products. The integration of diverse processing methods often yields better sensory outcomes, suggesting that multi-faceted approaches could meet consumer expectations more effectively [11].

Freezing extends the shelf-life of meat but causes physical and biochemical changes that reduce quality. Despite efforts over the past two decades, no single technique can fully address these issues, and the consumer perception of frozen meat remains negative. A review [143] explored novel freezing and thawing technologies, such as deep freezing and high pressure, and processing strategies to improve thawed meat quality and bridge the gap with fresh meat. While some approaches show promise in enhancing various meat quality traits, no single technology or strategy can be easily adopted by the meat industry to maintain all quality aspects of meat comparable to fresh equivalents, suggesting that further research needs to be carried out to enhance thawed meat quality.

Processing serves not only to meet the objective of preserving meat but also to enhance the acceptability and increase the variety of products offered to consumers. Moreover, processing can increase consumers’ acceptability of meat which is usually less acceptable, such as that of older or heavier animals, and non-traditionally eaten meats. While processed meat products are convenient, there is increasing awareness of the potential health risks associated with high consumption [144,145]. This has led to a demand for cleaner labels and healthier processing methods. Consumers prefer minimally processed meat because they associate it with higher quality and fewer additives.

Many studies have been conducted to evaluate the effect of modifying ingredients on the quality characteristics, specifically the sensory characteristics, of meat products to make them healthier and still accepted by consumers [146,147,148,149,150,151]. The mentioned modifications include reducing fat content, replacing healthier fats, using natural ingredients, and applying processing as ultrasounds to reduce or eliminate the effects of the modifications in the formulations.

The main findings of studies on consumer expectations, perceptions, and evaluations of diverse processed meat products are summarized in Table 3.

Consumers’ perceptions do not always align with the product’s real characteristics. A good marketing strategy from the meat and meat products’ industry, informing consumers about the products, is important to increase consumption.

## 6. Conclusions

The quality of meat and meat products is a subjective and dynamic concept aiming to please consumers. Every factor influencing consumer perception of quality is crucial. Achieving the desired quality sustainably requires considering all actors in the production chain, from production to consumption, including carcass and meat quality and their processing. Each step is vital to meet consumer demands for nutritious, healthy, and tasty meat products produced under sustainable and welfare conditions. This review highlights the impact of feeding and processing on meat and meat product quality, which can affect consumers’ perceptions and buying intentions. Information is fundamental to providing consumers with all the data to help them make intelligent purchases for their food consumption needs.

## Figures and Tables

**Table 1:** Effect of different feed sources in animal diets on meat quality.

Breed	Feed	Impact on Meat Quality	Reference
Lamb	Concentrates	Higher flavor, juiciness, and overall acceptability. With 1.5% concentrate feed, the lamb performed better in terms of the physicochemical, sensory, and instrumental color parameters of the lamb meat.	[8]
	Grape pomace	No changes in the sensory parameters. Higher total lipid levels, higher participation of polyunsaturated fatty acids (18:2n6 and CLA), and greater oxidative stability.	[22]
	Olive cake	Increased concentration of vitamin E in muscle and extended meat oxidative stability. Did not compromise animal growth, carcass weight, or muscle fat content.	[23]
Pork	Shredded acorns	Improvements in the fatty acid profiles away from SFAs towards MUFAs Slightly lower lean meat content and higher measured backfat thickness.	[24]
	Insect (*Tenebria molitor*)	Lower digestibility. Improvement in immunity and prevention of infection by pathogenic bacteria.	[25]
	Tomato pomace	Did not affect animal performance. Increased concentration of vitamin A in meat, reduced deposition of intramuscular fat.	[26]
	Olive cake	Increased muscle length. Did not affect the carcass characteristics or the meat and fat quality.	[27]
	Vitamin D_2_-enriched mushroom powder	Improved feed efficiency. Highest antioxidant activity and improved the overall color stability of fresh pork.	[28]
	Bergamot pulp	Greater oxidative stability. No effects from diet were observed on animal performance or on the fatty acid composition of the meat.	[29]
	Olive pomace acid oil	Negative impact on pork’s oxidative parameters. Color and overall acceptance of pork were not significantly affected by any of the diets.	[6]
Chicken	Plant polyphenolic compounds	Reduced lipid peroxidation during storage. Increased lightness and increased crude fat.	[30]
	Diets supplemented with proteins, fats, and carbohydrates	The dextrose supplement increased the content of dry matter, oleic acid, and amino acids. The casein supplement increased the content of dry matter, protein, and stearic acid.	[31]
	Garlic leaves	The treatment lowered the abdominal fat content lower than the control. Higher protein digestibility and metabolic energy (thereby increasing body weight).	[32]
	Sorghum grain	Did not affect the carcass, breast, and leg yield or organ size. Results indicated that this cereal could modify plasma lipids and improve some meat quality traits in broilers.	[33]
Goat	Hempseed meal	Its use did affect some measures of meat quality over the shelf-life. Various inclusion levels of this supplement have no impact on carcass characteristics.	[34]
Ducks	*Asytasia gangetica*	No significant differences were observed in growth performance of ducks. Improved nutrient intake and digestibility.	[35]
	Ad libitum beet pulp silage	The feeding approach had no adverse effects on most carcass or qualitative physicochemical characteristics of the muscles, except water-holding capacity in leg muscles. More efficient nutrient utilization.	[36]
Rabbits	Marine macroalgae products	Did not negatively affect the sensorial properties. Positively modified the fatty acid profile by increasing the proportion of omega 3.	[37]
	*Moringa oleifera* leaves	To obtain positive results, feed ratio should not exceed 700 g/kg of feed. Improved growth performance, feed conversion ratio and functional attributes of meat as compared to cowpea hay.	[38]

**Table 2:** Summary of studies on the effects of processing techniques on meat quality.

Type of Process	Process	Main Findings	Reference
Thermal	Dry-aging	Concentration of free amino acids. Improvement in meat palatability.	[46]
		Changes in meat color, water-holding capacity, tenderness, lipid oxidation, and protein degradation.	[47]
		Reduces Salmonella and E. coli levels but is ineffective against *Listeria monocytogenes* and *Yersinia enterocolitica.*	[48]
	Sous-vide cooking	Applied at low temperatures for a long time, it maintains meat’s nutritional composition and color. At moderate temperatures, sous-vide cooked meat retains its structure. Low flavor can be a drawback.	[50]
		Roasting in the oven before or after sous-vide cooking of lamb meat leads to a browner surface and a more intense cooked-meat flavor.	[51]
		The cooking temperature and time of sous-vide significantly influenced chicken breast’s physicochemical and palatability characteristics.	[54]
	Freezing	The freezing and thawing combinations did not cause remarkable changes in the quality parameters; rapid freezing, in the order of cryogenic freezing, individual quick freezing, and natural convention freezing, minimizes quality deterioration.	[59]
		Consumer perception is neither direct nor clear regarding the safety and technological benefits associated with cold technologies. More research is needed to explore the potential of these methods.	[58]
Non-thermal	High hydrostatic pressure	Used to enhance microbiological safety without high temperature. Maintaining organoleptic characteristics.	[63]
		Preserved nutritional value and shelf life extended without the use of preservatives or reduced additives.	[62,65]
	Irradiation	Effective in destroying pathogenic and spoilage microbes and improved organoleptic characteristics	[67]
		Not recommended for use with foods with a high-fat content as it accelerates lipid oxidation.	[73]
	Plasma technology	Can be applied to the disinfection of air, water, and food surfaces with low-cost investment.	[80,81]
Other	Chemical and bio-preservatives	Easily adapted to different food products to prevent oxidation, antimicrobials, and extend shelf life.	[89,95]

**Table 3:** Main findings of studies on consumer expectations, perceptions and evaluations of diverse processed meat products.

Product	Main Findings	Reference
Dry-aged grass-fed beef loins	Dry-aging has the potential to enhance the eating quality of low-marbled grass-fed beef without negatively impacting its microbial characteristics. Consumer survey data reveal that, although the market is small, there is a niche segment where consumers are willing to pay premium prices for dry-aged grass-fed beef.	[152]
Minced and burger meat (beef–pork or chicken–turkey) using different packaging (on trays, bulk).	Most respondents overestimated the fat content in various types of minced meat, as fat differences within a range of ±2% could not be detected. The color and appearance of the products were crucial for consumers, who placed little importance on the presence of additives. Unpackaged beef–pork meat was seen as more natural but also perceived as fattier and less healthy. Chicken–turkey meat was associated with healthiness and low-fat content but also with dislike, suggesting that additional processing should be carried out to improve appearance.	[153]
Long-term frozen storage of lamb meat	Consumers rated meat frozen and stored for 21 months as the lowest in terms of acceptability, while they preferred meat stored for 1 month. Despite this, all meats were considered “acceptable”. Interestingly, a third of consumers gave fresh meat the lowest acceptance after consumption, even though its visual appeal lasted 3 days longer than most thawed meats. Thawed and fresh meats were equally preferred when displayed for a short period, suggesting that concerns about thawed meat might need to be reconsidered.	[154]
Frozen meat	When consumers perceive frozen meat as having better nutritional content, appealing sensory qualities, and reasonable pricing, their attitudes towards these products improve, which in turn boosts their intention to purchase.	[155]
Gamma-irradiated ground beef	A consumer acceptance analysis was conducted on samples treated with 2.5 kGy of gamma irradiation. The results showed no significant differences between the irradiated and control samples. Consequently, 2.5 kGy was determined to be the optimal gamma-irradiation dose for reducing STEC in ground beef without affecting consumer acceptance.	[156]
Burger containing beef, pea, or algae protein	Participants in all three countries expected pea and algae burgers to be less tasty but healthier and more eco-friendly than beef burgers. These expectations were negatively impacted by higher meat commitment, negative attitudes towards vegetarian and vegan lifestyles, and higher food neophobia.	[157]
Processed meat products with reduced levels of nitrite	Consumers were generally unaware of nitrite in meat products but still showed positive attitudes and purchase intentions towards new processed meats. Purchase intention was positively linked to favorable attitudes, preference for natural additives, perceived harm of chemical additives, risk importance, innovativeness, awareness of nitrite, education, health interest, and frequency of processed meat consumption.	[144]
Low-fat content salami	Consumers showed favorable acceptance of reduced-fat salami, indicated by their willingness to pay more for it. This highlights that health awareness is a key factor in marketing cured meats.	[158]
Low-sodium salted meat	Regular sodium-salted meats were linked to health concerns and described as too salty, fatty, having a salty and strange taste, and contributing to high blood pressure. In contrast, low-sodium samples were associated with a good appearance, a metallic taste, and healthiness.	[159]
Nitrite-free cured pork loins	Producing nitrite-free cured loins is feasible if pathogen control is ensured, though the product may have a weaker color. However, consumers value sensory qualities beyond color, and the positive perception of an “additive-free” label can support the production of nitrite-free cured loins.	[160]

## Data Availability

The original contributions presented in this study are included in the article. Further inquiries can be directed to the corresponding author.
